# Sirolimus for the Treatment of Airway Obstruction due to Indolent T-Lymphoblastic Proliferation

**DOI:** 10.1155/2019/1724083

**Published:** 2019-12-09

**Authors:** Eric Moughames, Ana P. Kiess, Lee M. Akst, Antoine Azar

**Affiliations:** ^1^Department of Medicine, Johns Hopkins University School of Medicine, Baltimore, MD, USA; ^2^Department of Radiation Oncology and Molecular Radiation Sciences, Johns Hopkins University School of Medicine, Baltimore, MD, USA; ^3^Department of Otolaryngology-Head and Neck Surgery, Johns Hopkins University School of Medicine, Baltimore, MD, USA

## Abstract

**Introduction:**

Indolent T-lymphoblastic proliferation (iT-LBP) is a rare nonmalignant entity that presents as a proliferation of T-lymphoblasts. We report a first such case with a recurrent laryngeal obstruction presentation that was successfully controlled with Sirolimus.

**Case presentation:**

This is the case of a 29-year-old female who presented with a recurrent significant lymphoid hyperplasia in the adenoid and tongue base region as well as a right cervical lymph node. After repeated adenoidectomies and tonsillectomies, and based on pathological and clinical findings she was diagnosed with iT-LBP. Trials of radiotherapy and immunotherapy with cyclosporine and rituximab all failed to control the progression of the disease. Sirolimus was finally able to restrict the growth and improve her symptoms.

**Conclusion:**

While It-LBP does not usually require treatment, it is important to report cases in which treatment was crucial for the survival of the patient, and the effective role of Sirolimus in doing so, without any major adverse effects.

## 1. Introduction

Indolent T-lymphoblastic proliferation (iT-LBP) is a rare nonmalignant entity that presents as a proliferation of T-lymphoblasts most commonly involving, but not limited to, the nasopharynx and the oropharynx. It is distinguished from T-cell lymphoblastic lymphomas by several pathological and clinical features including a more indolent course. While there has been discussion of the pathology and most common presentations of iT-LBPs, there have been no reports on the role of effective immunotherapy for treating the disease. We report the case of an obstructing iT-LBP involving the nasopharynx, oropharynx, larynx and proximal trachea that was treated with Sirolimus with good result. 

## 2. Case Report

The patient is a 29-year-old female with a history of diabetes mellitus type 1 who presented to the clinic for evaluation of recurrent symptoms of sinusitis and a persistent nasopharyngeal mass. Her symptoms first started at the age of 12 with chronic nasal congestion, repetitive sinus infections and chronic cough. Her tonsils and adenoids were removed at the time, but her symptoms persisted. Between the ages of 13 to 15 she was found to have a recurrent adenoid mass and tonsillar regrowth. She underwent another adenoidectomy and tonsillectomy. Microscopic description of the specimen showed overall preservation of the architecture with follicular hyperplasia and mildly expanded paracortex with scattered immunoblasts. The follicles show polarized germinal centers and contain numerous tangible body macrophages. Immunohistochemistry showed that the interfollicular paracortical cells are positive for CD3, CD5, CD10, CD43, BCL-2, CD1a, CD7, CD4, CD8, and TdT. The tumor was also negative for clonally rearranged immunoglobulin heavy chain gene and negative for clonal T-cell receptor gamma chain gene rearrangement. Additionally, the patient was noted to have an enlarged right cervical lymph node. Due to concerns about malignancy she was hospitalized for a bone marrow biopsy that was deemed negative.

Over the following years the patient developed progressively worsening severe thick nasal drainage, rhinorrhea, frontal pressure and headaches, for which she presented to the clinic again at the age of 25. Her neck and sinus CT scan revealed maxillary sinus disease and significant lymphoid hyperplasia in the adenoid and tongue base region as well as a right cervical lymph node. She underwent a revision endoscopic sinus surgery and an adenoidectomy. Biopsy of the right-sided inflammatory process demonstrated an atypical T-cell lymphoid infiltrate, with a Ki-67 of 50–60%. She was then given a month of methylprednisolone (2 mg) taper and her cervical adenopathy diminished in size for a few weeks before it grew back along with fullness of the adenoid region, right posterolateral tongue asymmetry and lingual tonsil hypertrophy. She was given glycopyrrolate and saline nasal spray for her mucous secretions and was operated on again with removal of right lingual tonsillary tissue. Pathology of the tongue tissue demonstrated a predominantly atypical immature T-cell proliferation comprised of CD3-positive cells that co-express CD5, CD7, CD99, TdT, and CD117 with nodules of CD20 positive B-cells and scattered plasma cells. The atypical T-cells were also positive for CD4 and focal CD8. Immunostains for kappa and lambda showed no light chain restriction revealing that the plasma cells were polyclonal. Based on the clinical and pathological findings she was diagnosed with indolent T-lymphoblastic proliferation.

**Figure 1:**
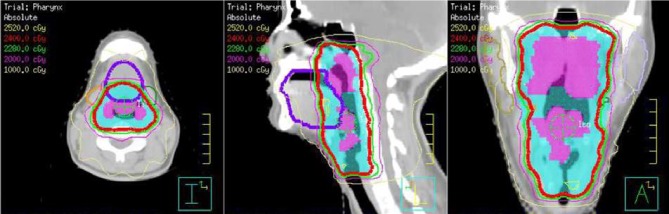
CT scan of the neck with contrast. Clinically involved tissues are shown in pink and the radiation planning target volume is shown in blue. The isodose lines show coverage of the target with 24 Gy and sparing of the salivary glands.

Upon follow up she was noted to have regrowth of the lymphoid tissue within the nasopharynx and oropharynx leading to new symptoms of dysphagia and an intermittent choking sensation due to fullness in the back of the nose and throat. Due to this regrowth, decision was made to treat her involved pharynx and larynx with 12 days of radiotherapy to a total dose of 2400 cGy as shown in Figure 1. Radiotherapy showed only partial improvement for 6 months and then symptoms progressed again and she was referred for an immunologic evaluation.

Prior to the initiation of immunotherapy, a full immunologic workup was done. This showed normal serum immunoglobulins and IgG subclasses, negative HIV and 16/23 protective post-immunization titers to Pneumovax. Tetanus and diphtheria IgG were low protective. Serum EBV, CMV and HBV viral loads were all negative. Decision was made to start her on 50 mg of cyclosporine BID which was later increased to 100 mg BID due to persistent symptoms. She was also asked to rate her symptoms in severity from 1 to 10 prior to the initiation of the drug (Table 1), in order to assess her response to the steroid-sparing immunosuppressants.

While on cyclosporine she reported episodes of recurrent sinus infections, progressive hoarseness and worsening dyspnea with increased work of breathing. On physical exam she had a regrowth of lymphoid tissue behind the left soft palate, increased lingual tonsil tissue which was partially obstructing her pharynx, and progressive airway distress with stridor. Laryngeal exam revealed fullness of the bilateral false vocal folds, fullness of the medial/inferior true vocal folds narrowing her airway, and irregular pink tissue of the proximal trachea all consistent with her proliferative T-cell process. The decision was made to provide definitive airway management through an awake tracheostomy with debridement of the excess laryngeal tissue.

With her airway safely secured, attention was returned to attempted medical management of her lymphoproliferative disorder. Due to lack of improvement on cyclosporine, a trial of 6 rituximab (100 mg) injections were administered. CT scan of the neck was performed following the 6 injections and showed no change. She was then started on Sirolimus, 2 mg daily.

After 2 months on Sirolimus the patient started to report improvement. She tolerated Sirolimus well with no side effects, had improved airflow through the nose and mouth, and denied any exercise limitations. Her symptoms scores have all improved (Table 1). There was also improvement noted on laryngoscopy in the subcordal/subglottic area, with stable disease in the nasopharyngeal and tongue base. She was thus downsized to a Montgomery cannula. 10 months following the initiation of Sirolimus, her nasopharynx was less obstructed and she was no longer dyspneic with exercise. The globus stopped bothering her as much as it previously did, and her sleeping improved. She had an MRI to delineate the oropharyngeal component and it showed some base of the tongue and right lateral pharyngeal wall involvement but none were obstructing. Laryngoscopy showed stable nasopharyngeal and tongue fullness and reduced burden of the subcordal/subglottic disease on the true vocal cords. Due to the remarkable improvement, decision was made for decannulation with close follow up at 3 months' intervals.

## 3. Discussion

Indolent T-lymphoblastic proliferation (iT-LBP) is a rare entity first described in 1999 by Velenkar et al. in a 33-year-old man with recurrent abnormal mass in the nasopharynx [1]. IT-LBPs were distinguished from lymphomas based on clinical and pathological findings [2–5]. While T-lymphoblastic lymphomas are considered high-grade lymphomas which if left untreated could spread rapidly with a high mortality rate, iT-LBPs have a more localized and slower course [2–5].

Because of the rarity of the disease and the lack of diagnostic guidelines, the diagnosis was based on pathological and clinical similarities to previous documented cases, after having ruled out T-cell lymphoma [6]. These findings include: The presence of T-cells expressing TdT and CD3, the presence of a heterogeneous population of immature T cells following the maturation pattern of normal thymocytes, small T-cells without significant morphological atypia, the lack of clonality and a clinically nonprogressive and indolent course. Most other presentations of the disease in the literature had a more stable course and did not necessitate pharmacotherapy or radiotherapy [7]. Since our patient had developed airway obstruction, necessitating a tracheostomy, an intervention was required. One similar case with airway obstruction due to recurrent enlargement of the oropharyngeal lymphoid tissue was reported, and chemotherapy was administered due to a preliminary misdiagnosis of malignant lymphoma [8]. While the CHOP-bleomycin (cyclophosphamide, doxorubicin, bleomycin) treatment only had a transient response in that patient, surgical debulking and chemotherapy with vincristine, prednisone, methotrexate and asparginase lead to the complete resolution of the mass [8]. Pathology reports in our case ruled out malignant causes of the lymphoid proliferation early in the manifestation of the disease and our treatment consisted of a trial of radiotherapy and then steroid sparing immunosuppressants targeting the lymphocytic proliferation when the former treatment showed limited results. The low dose of radiotherapy was chosen based on the typical sensitivity of lymphocytes to radiation [9, 10].

Because the earliest pathology reports demonstrated a T-cell origin of the lymphoid proliferation, cyclosporine was firstly used. Cyclosporine is a Calcineurin inhibitor that inhibits the activation of T-lymphocytes by inhibiting the transcription of IL-2 [11]. The failure of the response to cyclosporine can be explained by the fact that the T-cells were resistant to the inhibition of IL-2 by preventing the binding of the drug on the cytosolic protein cyclophillin. Due to the failure of the first drug targeting T-cells, a short trial of rituximab targeting B-cells was initiated. B-cells were also reported in the manifestation of the disease, and targeting these cells was thought to affect the progression of the disease when cyclosporine failed to show any change. Because this drug was also unsuccessful in showing any effect, the decision was made to target both T-cells and B-cells using Sirolimus, a more powerful immunosuppressant that acts as an mTOR inhibitor, which works by inhibiting the response to IL-2 and hence blocking both B-cell and T-cell activation using a different binding site [12]. The patient reported a marked reversal in symptoms and rhinolaryngoscopy showed a reduction in the size of the mass. The patient did not experience the commonly reported side effects of gastrointestinal disturbances, joint pain and infections attributed to Sirolimus. Her complete blood count has also remained unchanged.

In conclusion, we here report the first case of a recurrent obstructing iT-LBP that responded well to Sirolimus after unsuccessful control with surgical resections, radiotherapy and use of other immunosuppressants. This case report highlights the potential role of Sirolimus in the treatment of iT-LBP when therapy is necessary. Further trials of Sirolimus use on a large patient cohort can be useful in further exploring its role in treating iT-LBP, though this can be quite difficult given the rarity of the disease.

## Figures and Tables

**Table 1:** Diary of symptoms reported by the patient prior to the initiation of cyclosporine, 1-month post-cyclosporine, 3 and 10-months post-Sirolimus. Grades are assigned numbers 1–10, 10 being the worst and 1 being asymptomatic.

Main symptoms reported	Grades prior to immunotherapy	Grades after 1 month on cyclosporine	Grades after 3 months on sirolimus	Grades after 10 months on sirolimus
Post-nasal drainage	8	6, 7	3, 4	1, 2
Throat clearing cough	5, 6	5, 6	4	1
Mouth breathing	10	8	2	1, 2
Voice changes	8	5, 6	3	3
Swallowing problems (choking on food)	2, 3	2, 3	2, 3	1
